# Removing prophylactic antibiotics from pig feed: how does it affect their performance and health?

**DOI:** 10.1186/s12917-019-1808-x

**Published:** 2019-02-26

**Authors:** Alessia Diana, Laura A. Boyle, Finola C. Leonard, Ciaran Carroll, Eugene Sheehan, Declan Murphy, Edgar G. Manzanilla

**Affiliations:** 1 Pig Development Department, Teagasc Animal and Grassland Research and Innovation Centre, Moorepark, Fermoy, Co. Cork, Ireland; 2 School of Veterinary Medicine, UCD, Belfield, Dublin 4, Ireland; 3 Hilltop farms, Kilworth, Co. Cork, Ireland; 4 Makeway Limited Riverstown Business Park, Riverstown, Tramore, Co. Waterford, Ireland

**Keywords:** Antibiotic usage, Health, Performance, Pig, Prophylactic ban, Swine, Treatment

## Abstract

**Background:**

Antibiotics (AB) are an important tool to tackle infectious disease in pig farms; however some research indicates that their frequent mis/over-use may contribute to the development of antibiotic resistance and the WHO has declared that this issue should be addressed. Little is known about the long term consequences of withdrawing prophylactic AB from pig feed; hence we aimed to assess its effects on performance and health of pigs from weaning to slaughter.

Six batches of 140 pigs each were monitored on a commercial farm through the weaner and finisher stages to slaughter. In-feed antibiotics were not added to the feed for half of the pigs (NOI) and were added in the other half (ABI) within each batch for the whole weaner stage. Individual pigs in both treatments were treated with parenteral administrations if and when detected as ill or lame. Productive performance, parenteral treatments and mortality were recorded on farm and the presence of respiratory disease was recorded at slaughter. Pen was considered the experimental unit.

**Results:**

ABI pigs showed higher growth (*P* = 0.018) and feed intake (*P* = 0.048) than NOI pigs in the first weaner stage but feed efficiency was not affected (NOI = 1.48 vs. ABI = 1.52). Despite an initial reduction in performance, NOI pigs had similar performance in finisher stage (ADG: NOI = 865.4 vs. ABI = 882.2) and minimal effects on health compared to ABI pigs. No difference between treatments was found at the abattoir for the percentage of pigs affected by pneumonia, pleurisy, pleuropneumonia and abscesses (*P* > 0.05). Mortality rate was not affected by treatment during the weaner stage (*P* = 0.806) although it tended to be slightly higher in NOI than ABI pigs during the finisher stage (*P* = 0.099). Parenteral treatments were more frequent in NOI pigs during the weaner stage (*P* <  0.001) while no difference was recorded during the finisher stage (*P* = 0.406).

**Conclusions:**

These data suggest that the removal of prophylactic in-feed antibiotics is possible with only minor reductions in productive performance and health which can be addressed by improved husbandry and use of parenteral antibiotics.

## Background

Antibiotic (AB) usage in intensive livestock systems has been associated with antibiotic resistance (ABR) in some studies and the WHO (World Health Organization) has declared it a risk for both human and animal health [1]. The ban of AB as growth promoters was applied by the European Union in 2006 [2] and was important in reducing AB use. Ten years later, prophylactic AB are still used at high levels in many countries to sustain animal health and welfare [3, 4]. In order to promote more responsible use of AB, use should be assessed regularly, only allowed if strictly needed and alternative solutions should always be in place [5]. Such measures would help to reduce the selection pressure which contributes to the spread of ABR [5, 6].

The pig industry uses more medication (mg of active ingredient / population correction unit) than other livestock sectors, especially during the weaning period [7] when pigs face several challenges and stressors including changes in diet, separation from the sow and re-mixing. These changes stress the animals and compromise their immune system [8], making them more susceptible to infectious agents [9, 10]. The practice of prophylactic AB administered via the feed is an easy way of avoiding or reducing the risk of disease in weaned pigs. However, as such use is associated with a high likelihood of broad-spectrum usage or misuse [4, 11] it poses a threat for public health [12, 13]. The ban of in-feed AB usage proposed by the EU [14] includes provision for the removal of prophylactic in-feed AB use and the adoption of alternative strategies such as improved vaccinations or new management procedures [15–18]. Parenteral administration of AB would still be allowed, ensuring a more limited and targeted approach.

There are some published data suggesting that withdrawal of prophylactic AB is not necessarily associated with negative effects on production [19]. However, research is lacking on the long term consequences of withdrawing prophylactic AB on performance and health of pigs in commercial farms considering the whole production cycle. This information would be useful for veterinarians and policy makers in order to identify the most suitable practices to reduce the use of AB. Thus, the objective of this study was to assess the effect of removing prophylactic in-feed AB from the diet of weaner pigs on a commercial farm, allowing parenteral treatments as needed, on pig performance and health throughout the whole production cycle.

## Results

### On farm measurements

#### Production data, mortality and parenteral administration of antibiotics

ABI pigs had higher ADG (*P* = 0.018) and ADFI (*P* = 0.048) than NOI pigs during the first weaner stage which led to 2 kg non-significant difference in final body weight between treatments at the end of the second weaner (*P* = 0.218) and finisher (*P* = 0.483; Table 1) stages. There was no difference in ADG, ADFI and FCR between ABI and NOI pigs during the finisher stage (*P* > 0.05, Table 1). Mortality rate tended to be higher in NOI pigs than ABI pigs during the finisher stage (*P* = 0.099) but was not affected by treatment during the entire weaner stage (*P* = 0.806; Table 2). There was a difference between treatments in the total amount of parenteral administration of AB (sum of doses administered when pigs were detected to be lame and/or systemically ill) during the entire weaner stage, with a total of 25% vs. 13.8% of pigs treated for NOI and ABI pigs, respectively (*P* <  0.001). No difference between treatments was recorded during the finisher stage for total parenteral treatments (*P* = 0.406; Table 2). Data were also analysed according to the reason for treatment (i.e. lameness or systemic illness, Table 2). Parenteral treatments for lameness did not differ between treatments for weaner pigs but differed for finisher pigs (NOI = 18.7% vs. ABI = 13.1%, *P* = 0.036). Parenteral treatments for systemic illness differed for weaner (NOI = 23.6% vs. ABI = 12.4%, *P* < 0.001) but not for finisher pigs (*P* = 0.314).

**Table 1:** Production data. Average daily gain (ADG), average daily feed intake (ADFI), feed conversion ratio (FCR) and body weight (BW) for pigs provided with in-feed antibiotics (ABI) and for pigs with no in-feed antibiotics (NOI)

	First weaner stage^a^			Second weaner stage^a^			Finisher stage		
Variables	NOI	ABI	*P*-value	NOI	ABI	*P*-value	NOI	ABI	*P*-value
ADG g	402.2 ± 18.20	435.6 ± 13.03	0.018	711.0 ± 32.31	743.7 ± 42.58	0.774	865.4 ± 29.29	882.2 ± 29.29	0.893
ADFI g	584.6 ± 39.88	646.5 ± 28.83	0.048	1380.9 ± 29.29	1440.2 ± 60.09	0.589	1811.1 ± 31.09	1818.8 ± 36.92	0.984
FCR	1.48 ± 0.034	1.52 ± 0.032	0.483	1.95 ± 0.054	1.95 ± 0.045	0.944	2.10 ± 0.040	2.07 ± 0.038	0.853
BW Kg	21.9 ± 0.85	23.0 ± 0.70	0.032	41.4 ± 1.36	43.3 ± 1.40	0.218	99.4 ± 1.55	101.4 ± 1.91	0.483

Data are presented as means ± SEM (standard error of the mean)

^a^ First and second weaner stages data has already been published in Diana et al. (2017) [11]

**Table 2:** Mortality rate and parenteral administration of antibiotics for pigs provided with in-feed antibiotics (ABI) and for pigs without in-feed antibiotics (NOI)

	Weaner stage			Finisher stage		
Variables	NOI	ABI	*P*-value	NOI	ABI	*P*-value
Mortality rate%^a^	2.14	1.9	0.806	3.13	1.33	0.099
Injections tot %^b^	25	13.8	< 0.001	34.1	31.2	0.406
Injections lame%^1^	1.4	1.4	0.999	18.7	13.1	0.036
Injections sick%^2^	23.6	12.4	< 0.001	15.3	18.1	0.314

^a^ Percentage of pigs that died during weaner and finisher stages

^b^ Percentage of the animals treated based on the total number of parenteral administrations (in doses) recorded when pigs were considered systemically ill and lame, lame only^1^ and systemically ill only^2^ during weaner and finisher stages

Initial weaning and finishing BW were negatively associated with mortality rate and percentage of parenteral administrations of AB recorded during both stages (Table 3). Lighter weaner pigs were at greater risk of death (*P* = 0.056) and of being injected (*P* = 0.036) and lighter finishing pigs were at higher risk of being injected (*P* < 0.010; Table 3).

**Table 3:** Associations between initial body weight (BW) and the percentage of parenteral administrations of antibiotics and mortality rate and between final body weight and the percentage of pigs per pen affected by tail lesions across all pigs included in the study regardless of treatment

Variables	Initial BW^1^	*P*-value	Final BW^2^	*P*-value
	R^*^		R^*^	
Weaner stage				
Parenteral administrations %^a^	- 0.61	0.036		
Mortality rate %^b^	- 0.56	0.056		
Tail lesions %^c^			0.33	0.297
Finisher stage				
Parenteral administrations %^a^	- 0.54	0.002		
Mortality rate %^b^	- 0.17	0.384		
Tail lesions %^c^			- 0.52	0.018

^*^Correlation coefficients

^1^Body weight (kg) of pigs when moving to weaner and finisher stages

^2^Body weight (kg) of pigs at the end of the weaner and finisher stages

^a^Percentage of the animals treated based on the number of parenteral administrations (in doses) recorded when pigs were considered sick and/or lame during weaner and finisher stages

^b^Percentage of pigs that died during weaner and finisher stages

^c^Percentage of pigs per pen affected by tail lesions during weaner and finisher stages

#### Tail lesions

There was no difference in the percentage of pigs affected by tail lesions during the first (*P* = 0.168), second (*P* = 0.162) and finisher (*P* = 0.257) stages between ABI and NOI pigs (Table 4). At weaning, no association was detected between BW at the end of this stage and the percentage of pigs affected by tail lesions (*P* = 0.297), while at finishing final BW was negatively associated with percentage of pigs affected by tail lesions, with lighter pigs being at greater risk of having tail lesions (*P* = 0.018).

**Table 4:** Average percentage of pigs per pen affected by tail lesions for pigs provided with in-feed antibiotics (ABI) and for pigs with no in-feed antibiotics (NOI) during three time points of the production system

Production stages	NOI	ABI	*P*-value
First weaner %	5.9 ± 1.69	11.1 ± 3.47	0.215
Second weaner %	2.9 ± 1.34	8.2 ± 3.11	0.152
Finisher %	22.4 ± 7.35	13.6 ± 4.80	0.312

Data are presented as means ± SEM

### Slaughterhouse measurements

Less than 1% of heart, liver and lung condemnations were recorded at slaughter, therefore these data were not analysed. There were no differences in EP (*P* = 0.365) and pleurisy (*P* = 0.460) scores between ABI and NOI pigs (Table 5). Moreover, no difference between treatments was found for the percentage of pigs affected by EP (*P* = 0.945), pleurisy (*P* = 0.277), APP (*P* = 0.300) or abscesses (*P* = 0.142). The means and the corresponding standard error for each treatment are presented in Table 5.

**Table 5:** Results of EP (enzootic pneumonia) and pleurisy score and percentage of pigs with EP, pleurisy, lesions of APP (*Actinobacillus pleuropneumoniae*) and abscess recorded at slaughter for pigs with in-feed antibiotics (ABI) and without in-feed antibiotics (NOI)

Variables	NOI	ABI	*P*-value
EP score^1,a^	3.8 ± 6.75	5.0 ± 9.40	0.365
Pleurisy score^1,b^	0.5 ± 0.82	0.6 ± 0.83	0.460
EP %	40.8	41.3	0.945
Pleurisy %	33.7	41.3	0.277
APP % ^c^	0	1.1	0.300
Abscess % ^c^	0	2.2	0.142

^1^Data are presented as mean ± SE

^%^= percentage of pigs affected by EP (enzootic pneumonia), pleurisy, APP (*Actinobacillus Pleuropneumonia)* and abscess

^a^Scored according to the British Pig Executive, British Pig Health Scheme (2016)

^b^Scored using the Slaughterhouse Pleurisy Evaluation System (SPES; Dottori et al., 2007) from 0 = no lesions to 4 = severely extended lesions (at least 1/3 of both diaphragmatic lobes) and/or acute (exudation and abundant granulation tissue)

^c^Recorded as present or absent

## Discussion

The objective of this study was to assess the effect of removing prophylactic in-feed AB, but allowing parenteral treatments, on pig health and performance from weaning until slaughter. Although there is considerable variation in management and housing practices between Irish farms, the farm selected for this study was considered representative of the general situation in this country as it was a medium sized farm where the use of in-feed AB had become regular practice as an easy option to control background diseases.

Farmers benefit from the continuous use of in-feed prophylactic AB as it improves performance in a similar manner to the use of AB for growth promotion [11, 20]. Results from this study revealed that the use of AB at the weaner stage had indeed clear benefits for performance. Many studies on the use of in-feed AB in the weaning period have concluded that it is necessary to maintain performance as reviewed by Thacker [21]. However, this study showed that when the trial period was extended to the finisher stage where AB were not provided in pig feed, the differences in performance were not significant despite the fact that pigs with AB in their feed (ABI pigs) reached slaughter 2 kg heavier than pigs without (NOI pigs). The final heavier weight of ABI pigs was the result of higher ADG and ADFI during the first weaner stage. However, no difference in FCR was found between treatments showing that NOI pigs were as efficient as ABI pigs. Thus, the possible benefit of the extra sale weight is not so important economically as the amount of feed used is also less. Additionally, withdrawal of AB may also result in benefits for both consumers and farmers given the reduction in the amount of AB used.

During the weaner stage, NOI pigs had received double the parenteral treatments than ABI pigs. This difference might have been even more pronounced if the 2 groups of pigs had not shared the same room and air space; separation of the 2 groups might have reduced the infection pressure and the need for parenteral antibiotics in the ABI pigs. Nevertheless, the use of parenteral AB instead of in-feed AB still represents a very important reduction in the total use of AB per pig and probably allows for more accurate dosing of the AB, thus contributing less to AB resistance. On the other hand, the number of parenteral treatments in finisher pigs did not differ between treatments, showing that although the NOI pigs showed more clinical signs of illness during the weaning stage, this did not result in further consequences for the health of pigs during the finishing stage. There was also no difference between treatments in health indicators collected at the abattoir (EP, abscess, APP and pleurisy) or on tail lesions collected prior to slaughter. This also supports the hypothesis that withdrawal of in-feed medication did not compromise pig health which is in accordance with the results of other studies [15, 22, 23] where removal of in-feed AB did not result in health problems. However, looking at the different reasons for parenteral treatments, during the weaner stage the difference between groups was mainly because of systemic illness, whereas at finishing, it was because of lameness affecting NOI pigs. This difference may suggest some carryover effect of disease during the weaning stage. Infectious arthritis, often related to streptococcal infection, is a common cause of lameness in pigs [24, 25]. In-feed AB may provide protection against subclinical infections, sequestered in areas such as joints and that reappear later in the production cycle. However, given the multiple possible causes of lameness [26] further studies should be carried out to elucidate the reasons for the increase in lameness observed in pigs without AB in their diet. The higher mortality rate found at the finishing stage also supports the view that there may be a carryover effect from the weaner stage as no difference in mortality was detected during the weaner stages.

Finally, the relationships found between bodyweight, the percentage of parenteral treatments and mortality during both weaner and finisher stages reflect the strong link between weight of pigs at weaning and their susceptibility to disease. Other studies showed how lighter pigs had higher level of disease after weaning [27]. This suggests that body weight at weaning could be used as an early tool to monitor pigs that are considered at risk of disease later in life.

Before this study started, basic management practices such as stocking density, environmental control and enrichment were reviewed to make sure that removal of AB would not expose pigs to any unnecessary risks. Indeed, poor management conditions such as low levels of hygiene, unfavourable temperature or high stocking densities may facilitate the spread of pathogens [28]. Under such a scenario, in-feed AB show their greatest impact because they become an effective tool to control disease [29, 30]. Therefore, some simple adjustments were made but there was no financial investment to improve the housing or management of the pigs. Thus removal of in-feed prophylactic AB was possible without major risks for general pig health. Numerical differences in productive performance and mortality may be suggestive of some consequences of removing in-feed AB. However, in depth analysis of biosecurity, management and husbandry practices [31, 32] could provide sufficient information to allow correction of failures in these areas and prevention of such consequences. Additionally, injection of AB in clinically affected pigs may have contributed to the absence of health issues found in NOI pigs given that double the number of injections was administered during the weaner stages. It is also likely that greater vigilance on the part of the farm staff taking care of NOI pigs ensured that animals with early signs of clinical illness were promptly treated. This might also be expected in other farms where in-feed AB are removed.

The importance of developing new strategies and providing proper AB stewardship is underpinned by the possible contribution of AB use to the development of AB resistance [1]. Over/misuse may have detrimental implications for the length of treatment applied and for efficacy of the AB treatment. There was prolonged non-specific prophylactic treatment of weaner pigs on the study farm. Unfortunately, given the high use of in-feed AB in weaner pigs in some EU countries [7], it is likely that this farm is typical of many others both in Ireland and elsewhere. The type of withdrawal applied in this study resulted in a 97% reduction in overall AB use as measured in doses withdrawn from NOI pigs. This level of reduction may represent an advantage for both the finances of farmers and for public/animal health given the supposed contribution of AB use to the development of antibiotic resistance. However, if we consider the 2 kg lower weight at slaughter in NOI pigs, the saving on AB would not be enough to compensate this reduction in income. This is an important commercial issue to be considered when implementing legislation on this area.

## Conclusions

Overall, withdrawal of prophylactic in-feed AB did not result in major detrimental problems for performance and health and welfare of pigs. Untreated pigs were as efficient as pigs fed with AB although there were numerical reductions in production performance and a tendency towards higher mortality in the finisher stage. These results indicate that the removal of prophylactic in-feed AB, while still allowing the use of parenteral AB, is possible but will need some extra measures to be implemented to avoid loss of profit and impaired pig welfare.

## Methods

### Farm and animals

The study was carried out on a 300-sow farrow-to-finish commercial farm with a history of regular use of in-feed AB and included 6 weekly batches of Large White × Landrace crossbred pigs (840 pigs in total). The farmer expressed his willingness to cooperate with the intensive data collection required during the period of study which took place between September 2014 and February 2015. In order to comply with Council Directive 2008/120/EC, some changes in management such as an improved programme of environmental enrichment and a reduction in stocking density were introduced at the farm before the start of the study [11]. As per regular practice, piglets were first weaned at 28 ± 2 days of age and relocated to the first stage weaner accommodation where they spent 5 weeks. Thereafter pigs were moved to the second stage weaner accommodation for a further 4 weeks and finally to the finishing stage where the animals spent 8 to 11 weeks depending on the time they required to reach slaughter weight (approximately 110 kg). The farm had a clinical history of disease including episodes of meningitis and diarrhoea in the first stage weaner pigs and pleuropneumonia outbreaks both in the second stage weaners and finishers. Pigs were positive for porcine reproductive and respiratory syndrome virus (PRRSv), *Actinobacillus pleuropneumoniae*, *Mycoplasma hyopneumoniae* and swine influenza virus. In-feed prophylactic medication with sulfadiazine-trimethoprim (TMS) and therapeutic levels of ZnO (3000 ppm for 2 weeks) were used to address these clinical problems.

Weaner pigs were housed in rooms with four pens of 35 pigs each for the first stage and in rooms with eight pens of 17 pigs each for the second stage. Finisher pigs were housed in Trowbridge style pens (each pen is an independent room) of 21 to 23 pigs. Rooms for each stage had the same design and environmental control. Density was a minimum of 0.30 m^2^ per pig in the first stage weaner and a minimum of 0.40 m^2^ per pig in the second stage weaner. In the finisher stage density was a minimum of 0.65 m^2^ per pig. Pigs were housed on fully slatted floors, plastic for weaners with solid plastic panel pen divisions, and concrete for finisher pigs. Weaning facilities had an automatic temperature control system with fans in the ceiling and temperature was maintained at the recommended average of 26 °C for the first and 22.5 °C for the second stage [33]. The room was artificially illuminated from 0800 till 1700 h. The finisher facilities were naturally ventilated and lit with natural daylight. All pens had at least one nipple drinker with ad libitum water provided.

Pigs were weaned onto a commercial starter diet + electrolyte solution for a week and then moved to a weaner diet followed by a finisher diet, both of which were home milled. The weaner diet included corn (30%), barley (24.5%), soya bean meal 48% (23.5%), wheat (13.6%), lactofeed (Volac, Ireland; 2.5%) and soy oil (2.5%) (CP = 19.0%, DE = 14.4 MJ/kg, SID Lys = 1.15). The finisher diet included wheat (46%), barley (30%), soya bean meal 48% (18%), soy hulls (2%) and soy oil (1%) (CP = 16.3%, DE = 14.2 MJ/kg, SID Lys = 0.91). Pigs had ad libitum access to feed provided by a SPOTMIX liquid feeding system (Schauer Agrotronic GmbH, Prambachkirchen, Austria) and pens were furnished with environmental enrichment. This study was approved by the Teagasc Animal Ethics Committee (approval no. TAEC 40/2013) and the farm complied with Council Directive 2008/120/EC.

### Experimental design and treatment

Six weekly batches of 140 pigs were individually identified with an ear-tag and weighed at weaning (840 pigs in total, 9.2 ± 0.6 kg). During both weaning stages (for a total of nine weeks/five days a week), pigs received a diet where the in-feed AB (sulfadiazine-trimethoprim, 14.4 mg/kg BW/d) was randomly added to the diet (ABI) or not (NOI). In-feed AB were not administrated during the finisher stage, however pigs belonging to the same treatment (i.e. NOI or ABI) pens were kept together when moved to this production stage. This medication pattern was prescribed by the practising veterinarian 6 months before the study started in an empirical approach as per usual practice. Pigs were otherwise managed as per usual practice on the farm. Hence, it is important to highlight that pen composition changed between each stage according to regular farm management practices but the two populations of study pigs were kept separate throughout the production cycle. Due to these changes, the final number of pen replicates per treatment for the 6 batches was 12 for the first stage weaner, 24 for the second stage weaner and 18 pens for the finisher stage. During both weaning and finisher stages, pigs in both treatments were also treated with parenteral administration of amoxicillin (15 mg/kg BW) for 3 days by the farm staff if and when clinical signs of systematic illness (i.e. meningitis, severe respiratory disease, lethargy and failure to thrive and diarrhoea) or lameness were detected in individual animals.

### On farm measurements

#### Production data, parenteral administration of antibiotics and mortality

Performance measurements were recorded during all the stages. Each pen of pigs was weighed together at the end of the first and second weaner stages and before slaughter (i.e. end of the finisher stage) and the average pig weight was calculated. Daily feed intake of all pens was automatically recorded by the SPOTMIX liquid feeding system. The number of doses of parenteral injections of AB administered to pigs in each treatment was recorded daily by the farm staff during both the weaner and finisher stages. Two classes of parenteral treatments were recorded, lame and systemically ill. The proportion of pigs injected because of systemic illness or lameness in each treatment was calculated based on these records. Average daily gain (ADG), average daily feed intake (ADFI) and feed conversion ratio (FCR) were also calculated for all the stages. Number of animals that died was recorded daily by the farm staff during both weaner and finisher stages.

#### Tail lesions

A subset of 70 pigs per each weaning week (i.e. 35 per treatment) was selected for tail lesion assessment. Tail lesions were measured at three time points by one trained observer. The number of pigs per pen affected by tail lesions was recorded at the end of the first and second weaner stages. While prior to slaughter, tails were scored on a 5-point scale according to severity [34, 35]. The proportion of pigs per pen affected by tail lesions was calculated.

### Slaughterhouse measurements

At the abattoir pigs were stunned with CO_2_ and killed by exsanguination according to EU regulation. Enzootic pneumonia (EP) like lesions was scored according to the British Pig Executive (BPEX) Pig Health Scheme by one trained observer [36]. Additionally, presence or absence of *Actinobacillus pleuropneumoniae* (APP) lesions, abscesses and all instances of condemnations of heart, liver and lungs were recorded as per the decision of the acting veterinary inspector. Pleurisy was scored using the Slaughterhouse Pleurisy Evaluation System (SPES) [37] by one trained observer on a 3-point scale where 0 = no lesions; 1 = mild lesions, 2 = severe lesions. The proportion of pigs affected by EP, APP, abscess and pleurisy was also calculated.

### Statistical analysis

Data were analysed using SAS v9.4 (SAS Inst. Inc., Cary, NC). Alpha level for determination of significance was 0.05 and from 0.05 to 0.10 for trends. Data from the first and second weaner stages and the finisher stage were analysed separately to account for the change in pen composition between stages. Pen was always considered the experimental unit. Production data (ADG, ADFI and FCR) were analysed using general linear models; treatment was included as a fixed effect while initial mean body weight of the pen in each stage was included in the model as a covariate. In all stages, mortalities, injections and slaughter data (e.g. proportion of pigs affected by EP, APP, pleurisy and abscess) were analysed using Chi-square test while ANOVA was used to analyse EP and pleurisy scores between treatments. Tail lesion data were analysed using the general linear model in which treatment was included as a fixed effect. Results are presented as means ± SEM. Pearson correlations were calculated between initial body weight and mortality rate and between initial body weight and number of injections during the entire weaner (i.e. first and second) stage and during the finisher stage. Pearson correlations were also calculated between final body weights of weaner and finisher pigs and the corresponding proportion of pigs affected by tail lesions. Results are presented as the correlation coefficient (Rho) and the corresponding *P*-value.

## Abbreviations

AB: Antibiotics
ABI: Pigs with in-feed antibiotics
ABR: Antibiotic resistance
ADFI: Average daily feed intake
ADG: Average daily gain
ANOVA: Analysis of variance
APP: *Actinobacillus* pleuropneumoniae
BPEX: British Pig Executive
BW: Body weight
CP: Crude protein
DE: Digestible energy
EP: Enzootic pneumonia
EU: European Union
FCR: feed conversion ratio
NOI: Pigs without in-feed antibiotics
PRRSv: Porcine reproductive and respiratory syndrome virus
SEM: Standard error of the mean
SPES: Slaughterhouse Pleurisy Evaluation System
TMS: Sulfadiazine-trimethoprim

## References

[1] World Health Organization. Antimicrobial resistance: global report on surveillance. http://www.who.int/drugresistance/documents/surveillancereport/en/ (2014).

[2] European Commission. Ban on antibiotics as growth promoters in animal feed enters into effect. Press Release Database. 2005; http://europa.eu/rapid/press-release_IP-05-1687_en.htm.

[3] Aarestrup F (2012). Get pigs off antibiotics. Nature..

[4] Callens B, Persoons D, Maes D, Laanen M, Postma M, Boyen F (2012). Prophylactic and metaphylactic antimicrobial use in Belgian fattening pig herds. Prev Vet Med..

[5] World Health Organization. WHO Global Principles for the containment of antimicrobial resistance in animals intended for food: report of a WHO consultation with the participation of the Food and Agriculture Organization of the United Nations and the Office International des Epizooties, Geneva, Switzerland 5–9 June 2000. http://www.who.int/iris/handle/10665/68931 (2000).

[6] Codex Alimentarius Commission (CAC) – Code of practice to minimize and contain antimicrobial resistance (CAC/RCP 61–2005). CAC, Rome. http://www.fao.org/fao-who-codexalimentarius/codex-texts/codes-of-practice/en/ (2005).

[7] EMA, EFSA (2017). EMA and EFSA joint scientific opinion on measures to reduce the need to use antimicrobial agents in animal husbandry in the European Union, and the resulting impacts on food safety (RONAFA). The EFSA Journal.

[8] Campbell JM, Crenshaw JD, Polo J (2013). The biological stress of early weaned piglets. J Anim Sci Biotechno..

[9] Zoric M, Stern S, Lundeheim N, Wallgren P (2003). Four-year study of lameness in piglets at a research station. Vet Rec.

[10] Calderón Díaz JA, Boyle LA, Diana A, Leonard FC, Moriarty JP, McElroy MC (2017). Early life indicators predict mortality, illness, reduced welfare and carcass characteristics in finisher pigs. Prev Vet Med..

[11] Diana A, Manzanilla EG, Calderón Díaz JA, Leonard FC, Boyle LA (2017). Do weaner pigs need in-feed antibiotics to ensure good health and welfare?. PLoS One.

[12] Desrosiers R. Why we should reduce antibiotic usage and ways to do it. In: Abstracts of the 13th London swine conference: managing for production. London, Ontario, Canada, 27-28 March 2013. London: Swine Conference; 2013. p. 105–16.

[13] Laxminarayan R, Duse A, Wattal C, Zaidi AK, Wertheim HF, Sumpradit N (2013). Antibiotic resistance - the need for global solutions. Lancet Infect Dis.

[14] Anonymous (2016). MEPs vote to ban prophylactic use of antibiotics in animals. Vet Rec..

[15] Wierup M (2001). The Swedish experience of the 1986 year ban of antimicrobial growth promoters, with special reference to animal health, disease prevention, productivity, and usage of antimicrobials. Microb Drug Resist.

[16] Stein HH (2002). Experience of feeding pigs without antibiotics: a european perspective. Anim Biotechnol.

[17] European Commission. EIP-AGRI Focus Group 'Reducing antibiotic use in pig farming' Final Report. https://ec.europa.eu/eip/agriculture/en/publications/eip-agri-focus-group-reducing-antibiotics-pig (2014).

[18] Sjölund M, Postma M, Collineau L, Lösken S, Backhans A, Belloc C (2016). Quantitative and qualitative antimicrobial usage patterns in farrow-to-finish pig herds in Belgium, France, Germany and Sweden. Prev Vet Med.

[19] Postma M, Vanderhaeghen W, Sarrazin S, Maes D, Dewulf J (2017). Reducing antimicrobial usage in pig production without jeopardizing production parameters. Zoonoses Public Hlth.

[20] Aarestrup FM (2005). Veterinary drug usage and antimicrobial resistance in Bacteria of animal origin. Basic Clin Pharmacol Toxicol.

[21] Thacker PA (2013). Alternatives to antibiotics as growth promoters for use in swine production: a review. J Anim Sci Biotechno.

[22] Laine T, Yliaho M, Myllys V, Pohjanvirta T, Fossi M, Anttila M (2004). The effect of antimicrobial growth promoter withdrawal on the health of weaned pigs in Finland. Prev Vet Med..

[23] Alban L, Dahl J, Andreasen M, Petersen JV, Sandberg M (2013). Possible impact of the “yellow card” antimicrobial scheme on meat inspection lesions in Danish finisher pigs. Prev Vet Med..

[24] Jørgensen B, Arnbjerg J, Aaslyng M (1995). Pathological and radiological investigations on Osteochondrosis in pigs, associated with leg weakness. J Vet Med A..

[25] Kirk RK, Svensmark B, Ellegaard LP, Jensen HE (2005). Locomotive disorders associated with sow mortality in Danish pig herds. J Vet Med A.

[26] Smith B (1988). Lameness in pigs associated with foot and limb disorders. In Practice.

[27] Calderón Díaz JA, Diana A, Boyle LA, Leonard FC, McElroy M, McGettrick S (2017). Delaying pigs from the normal production flow is associated with health problems and poorer performance. Porcine Health Manag..

[28] Kil DY, Stein HH (2010). Invited review: management and feeding strategies to ameliorate the impact of removing antibiotic growth promoters from diets fed to weanling pigs. Can J Anim Sci.

[29] Roura E, Homedes J, Klasing KC (1992). Prevention of immunologic stress contributes to the growth-permitting ability of dietary antibiotics in chicks. J Nutr.

[30] Cromwell GL (2002). Why and how antibiotics are used in swine production. Anim Biotechnol.

[31] Postma M, Backhans A, Collineau L, Loesken S, Sjölund M, Belloc C (2016). Evaluation of the relationship between the biosecurity status, production parameters, herd characteristics and antimicrobial usage in farrow-to-finish pig production in four EU countries. MINAPIG consortium. Porcine Health Manag.

[32] Collineau L, Rojo-Gimeno C, Léger A, Backhans A, Loesken S, Nielsen EO (2017). Herd-specific interventions to reduce antimicrobial usage in pig production without jeopardising technical and economic performance. Prev Vet Med..

[33] FAWAC. Code of practice for the welfare of pigs. Farm Animal Welfare Advisory Council. Nov 2009. Available from: http://www.fawac.ie/media/fawac/content/publications/animalwelfare/CodePracticePigWelfare.pdf.

[34] Kritas S, Morrison RB (2007). 2007. Relationships between tail biting in pigs and disease lesions and condemnations at slaughter. Vet Rec..

[35] Teixeira DL, Harley S, Hanlon A, O’Connell NE, More SJ, Manzanilla EG (2016). Study on the association between tail lesion score, cold carcass weight, and viscera condemnations in slaughter pigs. Front Vet Sci.

[36] British Pig Health Scheme. BPHS Scoring System 2016. 2016. https://pork.ahdb.org.uk/health-welfare/health/safe-traceable-pork/pig-health-scheme/.

[37] Dottori M, Nigrelli A, Bonilauri P, Merialdi G, Gozio S, Cominotti F (2007). Proposta di un nuovo sistema di punteggiatura delle pleuriti suine in sede di macellazione La griglia S.P.E.S. (Slaughterhouse Pleuritis Evaluation System). Large Anim Rev.

